# Supplementary material for: Competing climate feedbacks of ice sheet freshwater discharge in a warming world
Source: Nat Commun. 2024 Jun 18;15:5178. doi: 10.1038/s41467-024-49604-3 (PMC11189430; doi:10.1038/s41467-024-49604-3)
Supplement: Supplementary file 1 — Supplementary Information [file 41467_2024_49604_MOESM1_ESM.pdf]

Competing climate feedbacks of ice sheet freshwater discharge in a warming world
Supplementary Information

Dawei Li[1,2,3*], Robert M. DeConto[4], David Pollard[4,5], Yongyun Hu[6]

[1]Key Laboratory of Polar Ecosystem and Climate Change, Ministry of Education; and School of Oceanography, Shanghai Jiao Tong University, Shanghai, 200030, China.
[2]Shanghai Frontiers Science Center of Polar Science, Shanghai Jiao Tong University, Shanghai, 200030, China.
[3]Key Laboratory for Polar Science, Polar Research Institute of China, Ministry of Natural Resources, Shanghai, 200136, China.
[4]Department of Earth, Geographic, and Climate Sciences, University of Massachusetts Amherst, Amherst, 01003, Massachusetts, United States.
[5]Earth and Environmental Systems Institute, Pennsylvania State University, University Park, 16802, Pennsylvania, United States.
[6]Laboratory for Climate and Ocean-Atmosphere Studies, Department of Atmospheric and Oceanic Sciences, School of Physics, Peking University, Beijing, 100871, China.

*Corresponding author(s). E-mail(s): lidavvei@sjtu.edu.cn;

Supplementary Table 1: List of experiments

	Experiment name	*Length (year)*	*Number of simulations*	*Description*
0	UVic_PI	5,000	1	Preindustrial simulation with pCO_2 = 280 ppm and present-day ice sheet geometry.
1	UVic_2xCO2	1,500	3	Abrupt doubling pCO_2 from 280 ppm to 560 ppm.
2	UVic_4xCO2	1,500	3	Abrupt quadrupling pCO_2 from 280 ppm to 1120 ppm.
3	GIS_TPDD	10,000	11	GIS tuning simulations with CERA-20C 1901-1920 and WOA2018 1981-2010 climatology. TPDD ranges from 0 °C to -5 °C.
4	AIS_OMF	1	10	AIS tuning simulations with WOA2018 1981-2010 climatological mean ocean temperatures and OMF ranging from 1 to 5.
5	AIS_INV	100,000	1	AIS inverse simulation with CERA20C 1901-1920 and WOA2018 1981-2010 climatology to calculate basal sliding coefficients for the Antarctic bed.
6	PI_FWC	5,000	1	Coupled ISM-CM simulation with no interactive ice sheet FWF.
7	PI_FWA	5,000	1	Same as 6 but with interactive Antarctic FWF.
8	PI_FWG	5,000	1	Same as 6 but with interactive Greenland FWF.
9	PI_FWAG	5,000	1	Same as 6 but with interactive FWF for both Greenland and Antarctica.
10	SSP119_FWAG	650	3	Coupled ISM-CM simulation under historical-SSP1-1.9 with interactive FWF for both Greenland and Antarctica. simulations are initiated from year 4,000 of Exp. 9. Each suite contain 3 members with an ECS of 3.0, 4.0, and 5.6 °C, respectively.
11	SSP126_FWAG	650	3	Same as Exp. 10 but under SSP1-2.6.
12	SSP245_FWAG	650	3	Same as Exp. 10 but under SSP2-4.5.
13	SSP370_FWAG	650	3	Same as Exp. 10 but under SSP3-7.0.
14	SSP460_FWAG	650	3	Same as Exp. 10 but under SSP4-6.0.
15	SSP585_FWAG	650	3	Same as Exp. 10 but under SSP5-8.5.
16	SSP245_ECS4.0_FWC	650	10	Coupled ISM-CM simulation under historical-SSP1-1.9 with an ECS of 4.0 °C. Each suite contain 10 ensemble simulations initiated from restart files from Exp. 9, corresponding to year 3100 to 4000 with a 100-year interval. The coupled model is configured with no interactive FWF from either ice sheet.
17	SSP245_ECS4.0_FWA	650	10	Same as Exp. 16 but with interactive AIS FWF.
18	SSP245_ECS4.0_FWG	650	10	Same as Exp. 16 but with interactive GIS FWF.
19	SSP245_ECS4.0_FWAG	650	10	Same as Exp. 16 but with interactive FWF from both Antarctica and Greenland.
20	SSP585_ECS5.6_FWC	650	10	Same as Exp. 16 but under SSP5-8.5 with an ECS of 5.6 °C.
21	SSP585_ECS5.6_FWA	650	10	Same as Exp. 17 but under SSP5-8.5 with an ECS of 5.6 °C.
22	SSP585_ECS5.6_FWG	650	10	Same as Exp. 18 but under SSP5-8.5 with an ECS of 5.6 °C.
23	SSP585_ECS5.6_FWAG	650	10	Same as Exp. 19 but under SSP5-8.5 with an ECS of 5.6 °C.
24	SSP585_ECS5.6_NOCF	650	4	Coupled ISM-CM simulations with cliff failure turned off and four configurations of ice sheet FWF: FWC, FWA, FWG, and FWAG. Simulations are initiated from year 4000 of Exp. 9 and CO_2 follows SSP5-8.5 with an ECS of 5.6 °C.

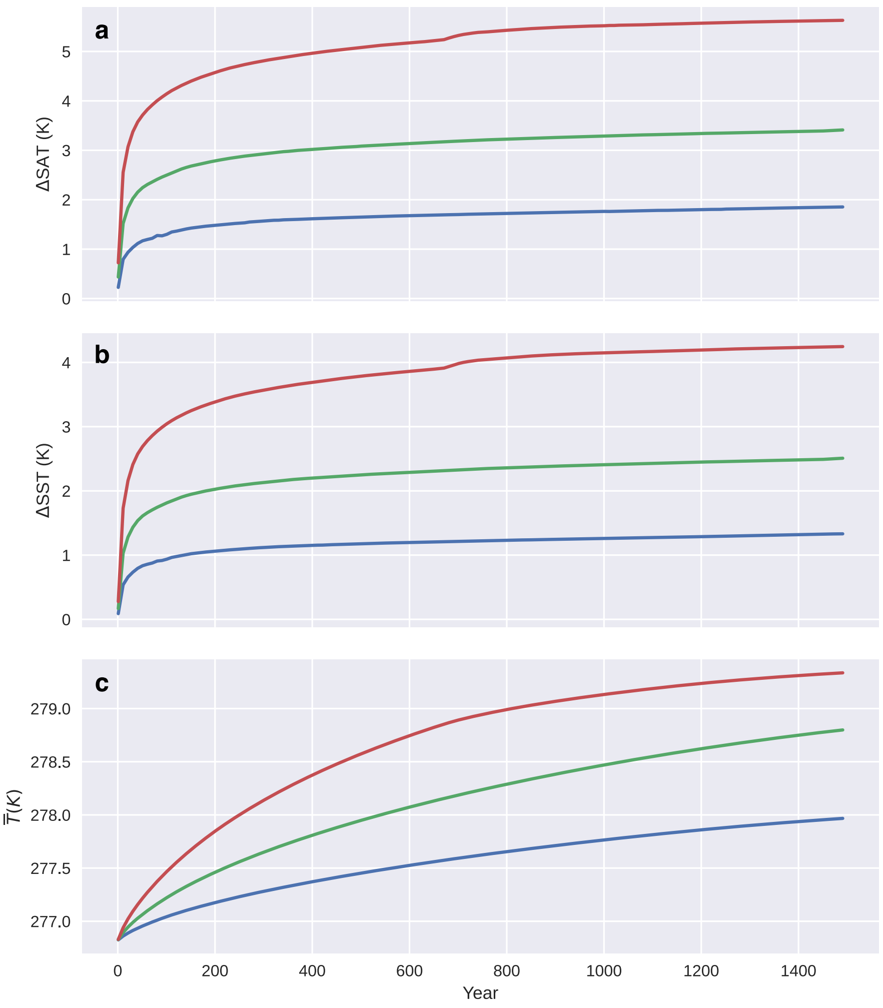

Supplementary Fig. 1: Results from abrupt-2xCO2 experiments with scaled CO_2 concentrations to emulate climate models with alternative Equilibrium Climate Sensitivities (ECS). Top to bottom panels show changes in **a** global mean surface air temperature (SAT), **b** global mean sea surface temperature (SST), and **c** global mean ocean temperature ($\bar{T}$). Each panel shows three emulated ECSs: a low ECS of 1.8 °C in blue, the native UVic-ESCM ECS of 3.4 °C in green, and a high ECS of 5.6 °C in red.

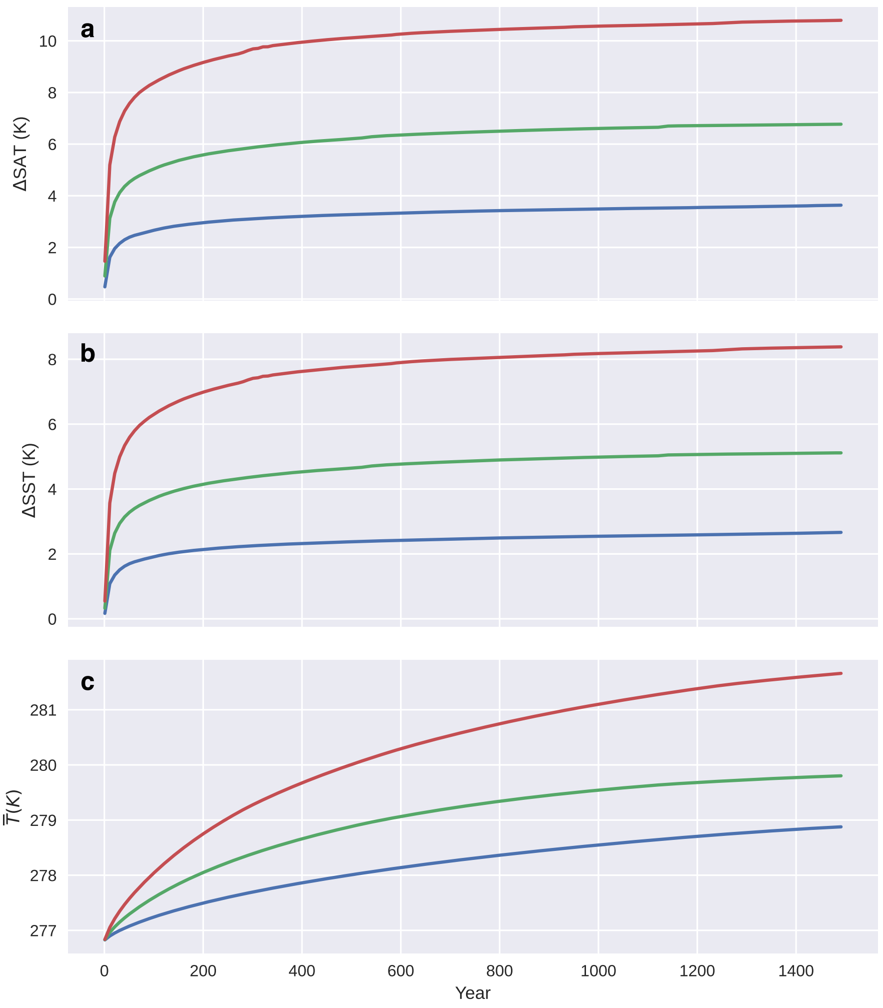

Supplementary Fig. 2: Same as Supplementary Fig. 1 but for abrupt-4xCO2 experiments.

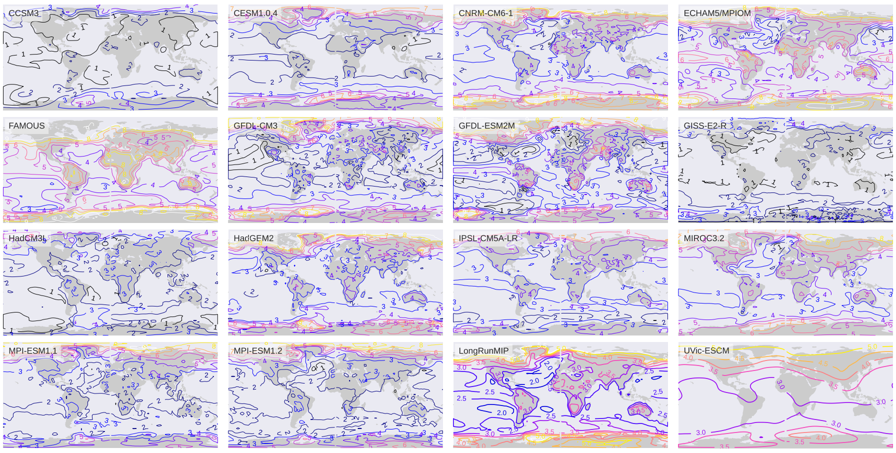

Supplementary Fig. 3: Maps of amplification factors in annual surface air temperature (SAT) with doubled atmospheric CO_2 level. The (dimensionless) amplification factor is defined as the change in SAT (ΔSAT) at each grid point with respect to its preindustrial condition divided by the change in GMSAT (ΔGMSAT) when the climate reaches a quasi-equilibrium after doubling pCO_2: AF $= \Delta\text{SAT}/\Delta\text{GMSAT}$. Panels show results from 14 individual LongRunMIP climate models, LongRunMIP multi-model-mean, and UVic-ESCM. Results for GFDL models are based on the 1pct-2xCO2 experiment, while for other models ΔSAT are from the abrupt-4xCO2 experiment but multiplied by a factor of 0.5.

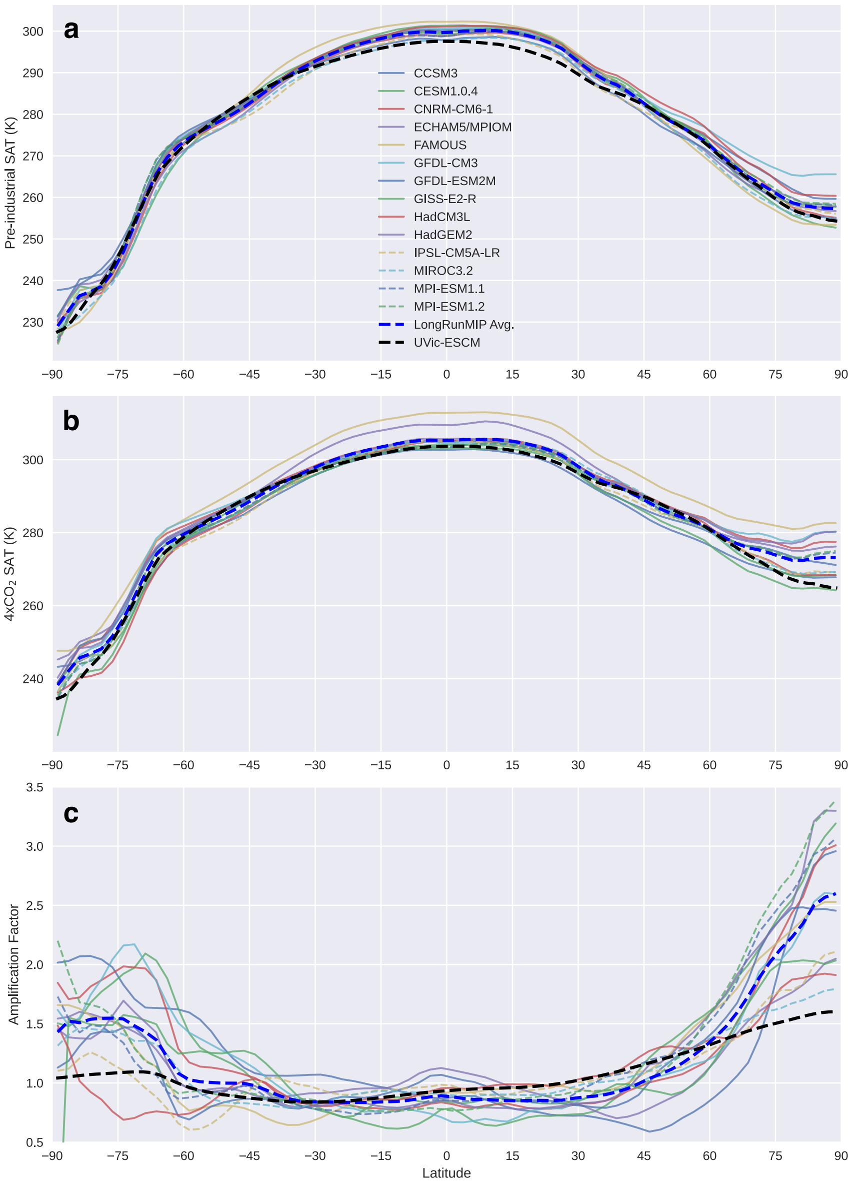

Supplementary Fig. 4: Zonal-mean amplification factors for SAT with doubled atmospheric $p\mathrm{CO}_2$.

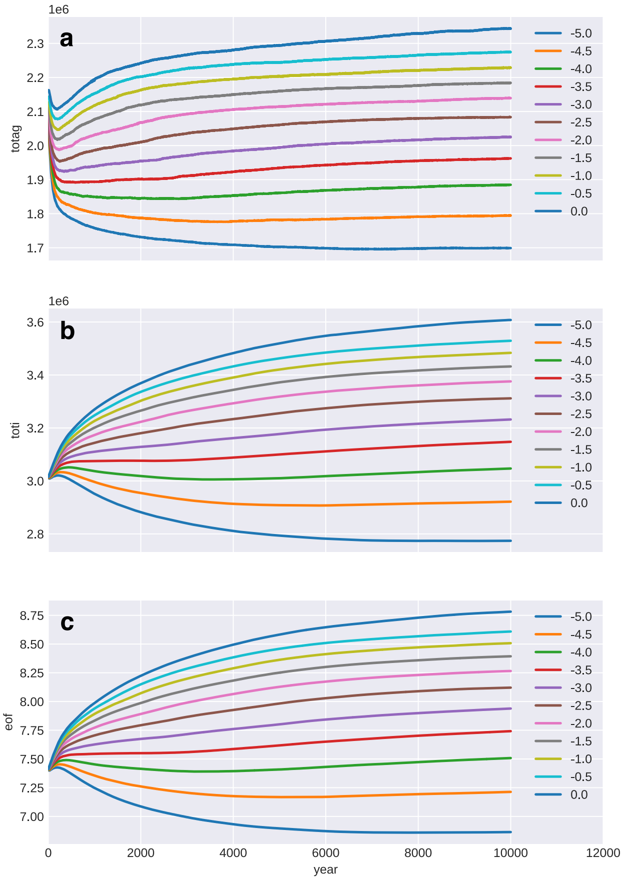

Supplementary Fig. 5: Results from GIS_TPDD tuning experiments. Panels from top to bottom show ice sheet area (totag, km^2), ice volume (toti, km^3), and sea level equivalent (eof, m) of the Greenland Ice Sheet. Temperature offset for the Positive Degree Day scheme (TPDD) ranges from 0 °C to -5 °C, 0.5 °C apart in a series of 11 simulations.

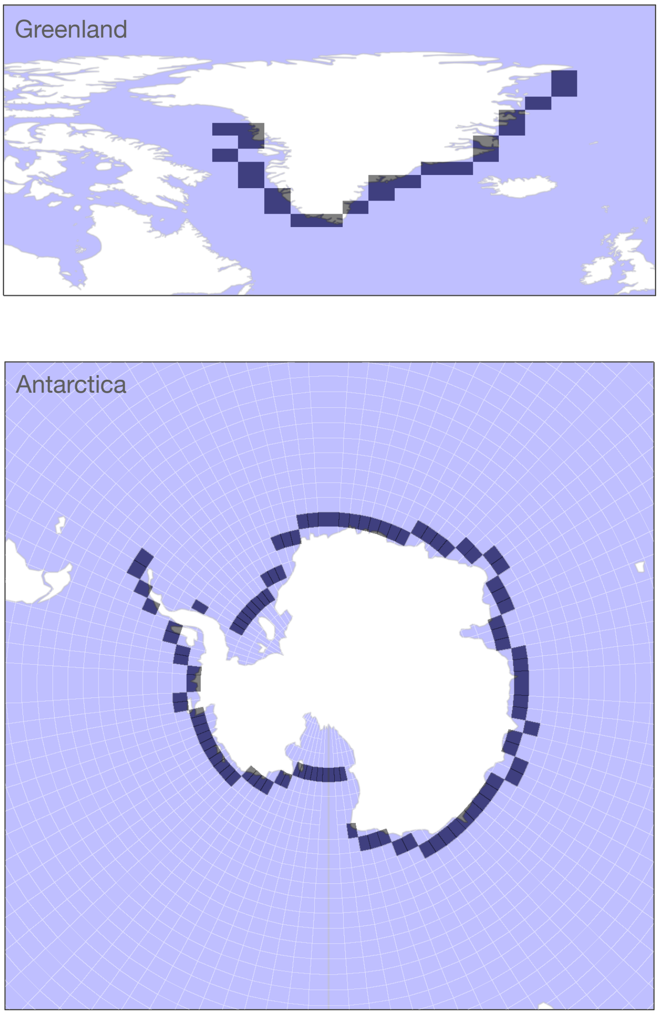

Supplementary Fig. 6: Coastal grid cells of Greenland and Antarctica in UVic-ESCM for applying ice sheet freshwater flux in the coupled ice sheet-climate model.

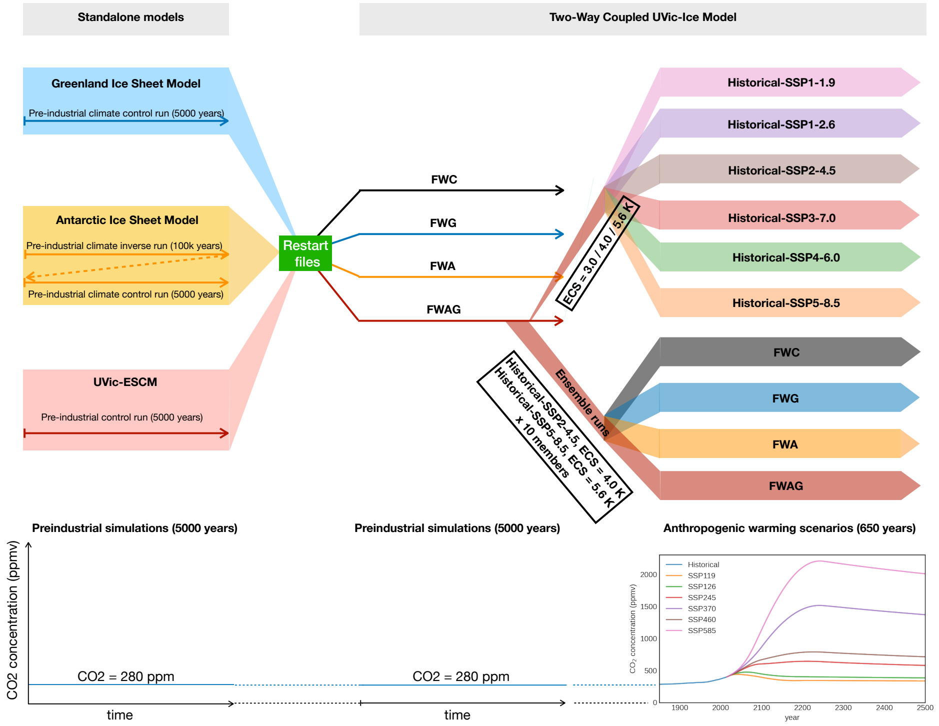

Supplementary Fig. 7: Schematics of numerical experiments carried out in this study.

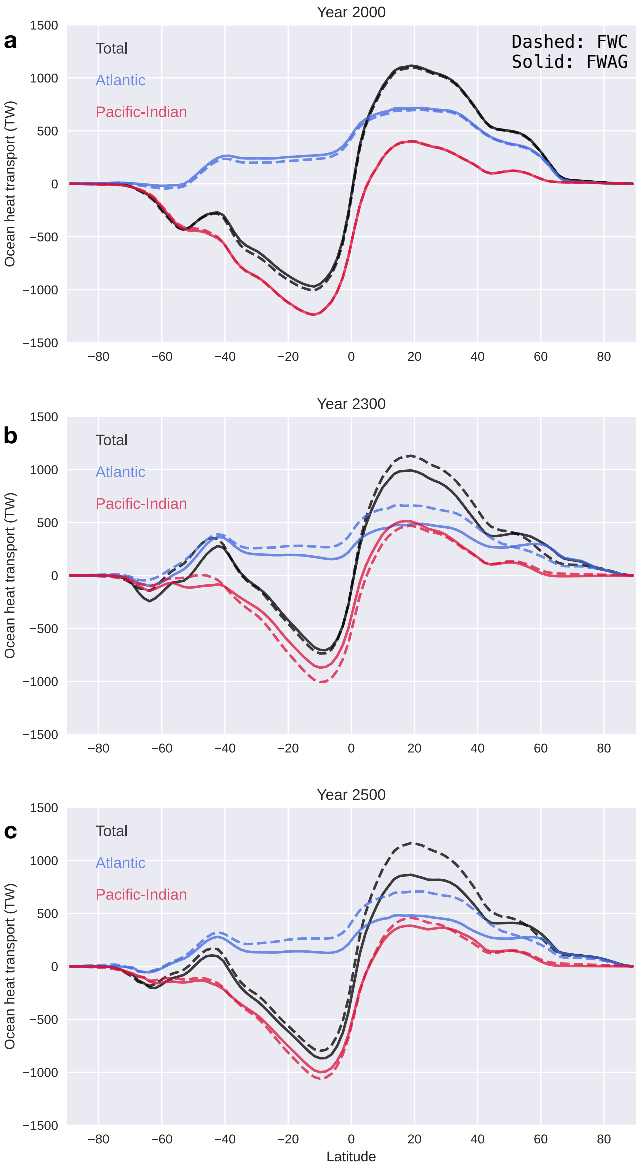

Supplementary Fig. 8: Meridional ocean heat transport in experiments without interactive ice sheet freshwater flux (FWC, dashed lines) and interactive freshwater flux from both ice sheets (FWAG, solid lines) for the coupled model simulations with an emulated ECS of 5.6 °C in the SSP5-8.5 scenario. Black, blue, and red lines represent total heat transport and those contributed by the Atlantic Ocean and Pacific-Indian Oceans respectively. Panels a, b, and c show snapshots at the years 2000, 2300 and 2500 respectively.

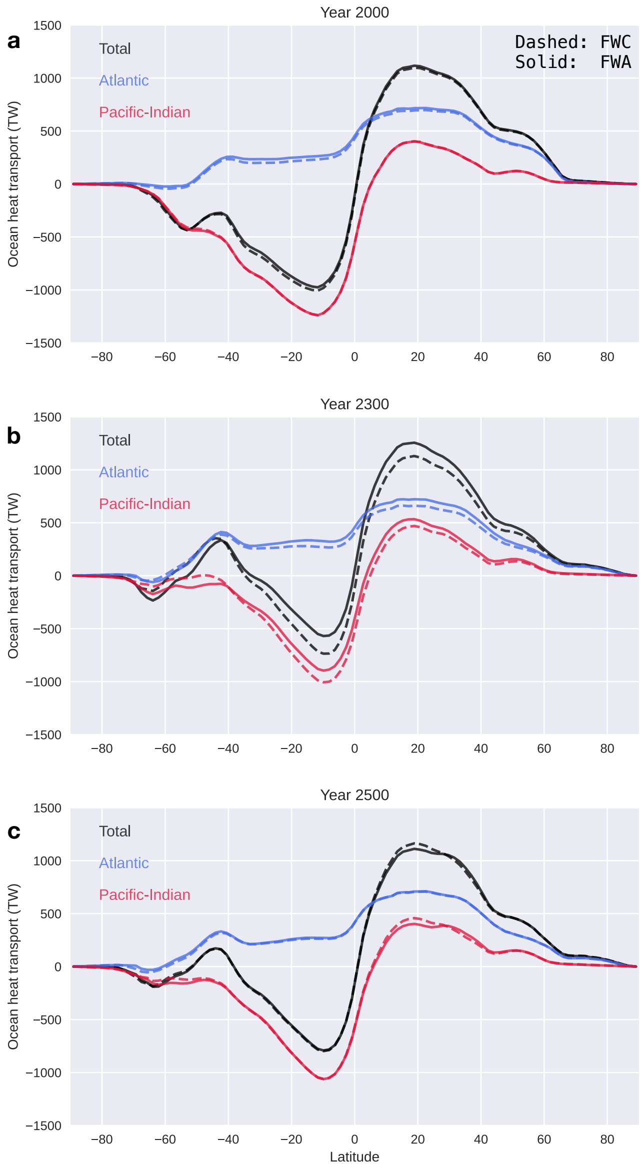

Supplementary Fig. 9: Same as Supplementary Fig. 8 but solid lines are for experiments with interactive Antarctic freshwater flux only (FWA). Panels a, b, and c show snapshots at the year 2000, 2300 and 2500 respectively.

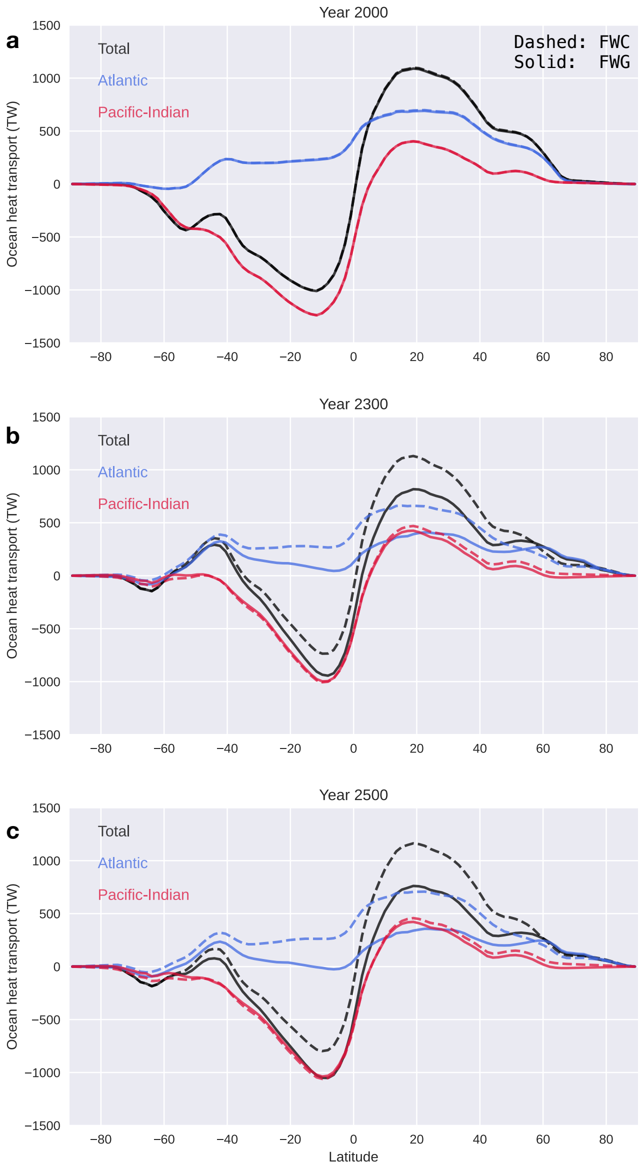

Supplementary Fig. 10: Same as Supplementary Fig. 8 but solid lines are for experiments with interactive Greenland freshwater flux only (FWG). Panels a, b, and c show snapshots at the year 2000, 2300 and 2500 respectively.

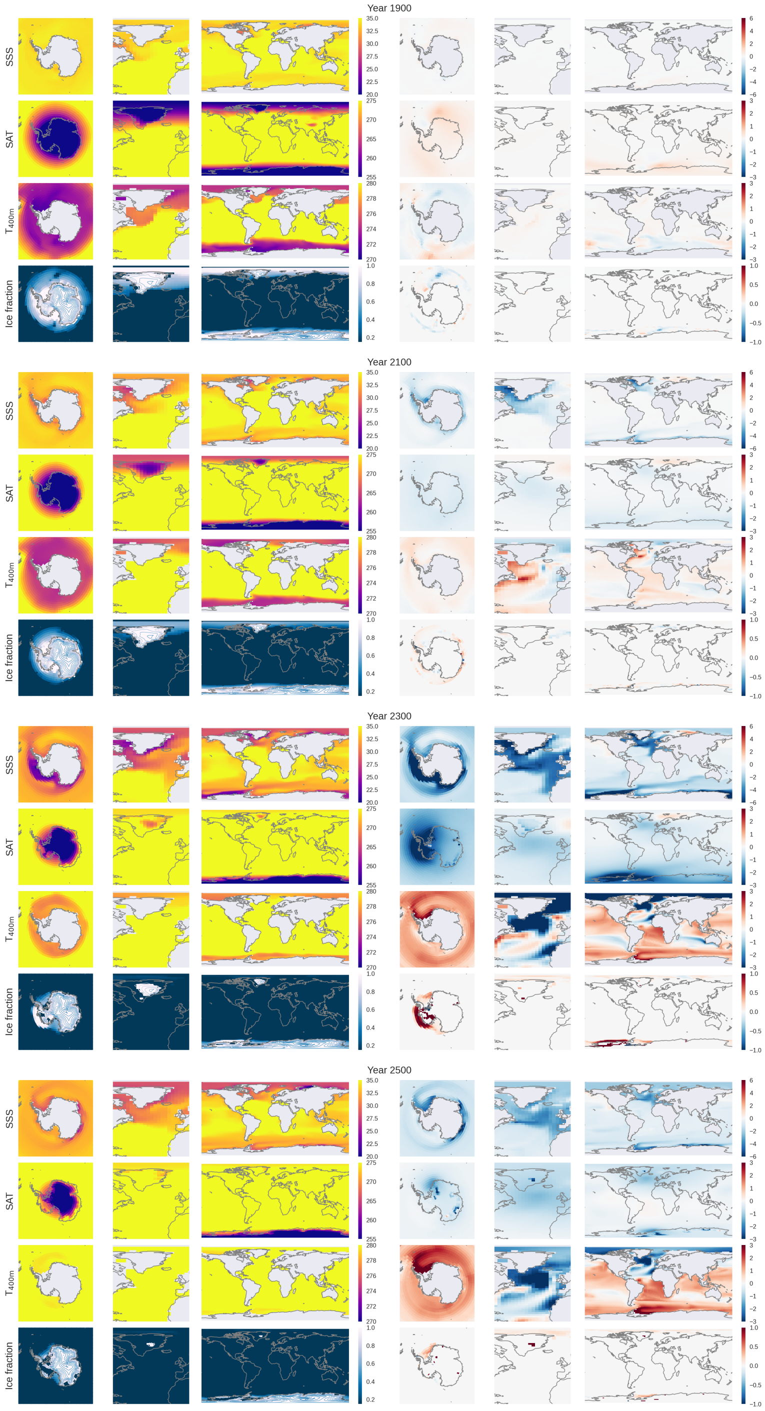

Supplementary Fig. 11: Snapshots of maps of selected variables in year 1900, 2100, 2300, and 2500. In each panel group, rows from top to bottom show sea surface salinity (SSS), surface air temperature (SAT), subsurface ocean temperature at 400 m (T_{400m}), and ice fraction (blue contours show ice surface elevation with an interval of 200 m); The left half display variables from coupled ice sheet-climate model simulation with the FWAG (interactive freshwater flux for both ice sheets) configuration under historical-SSP5-8.5 and an Equilibrium Climate Sensitivity (ECS) of 5.6 °C, while the right half show differences in these variables between FWAG and the simulation without interactive ice sheet freshwater flux.

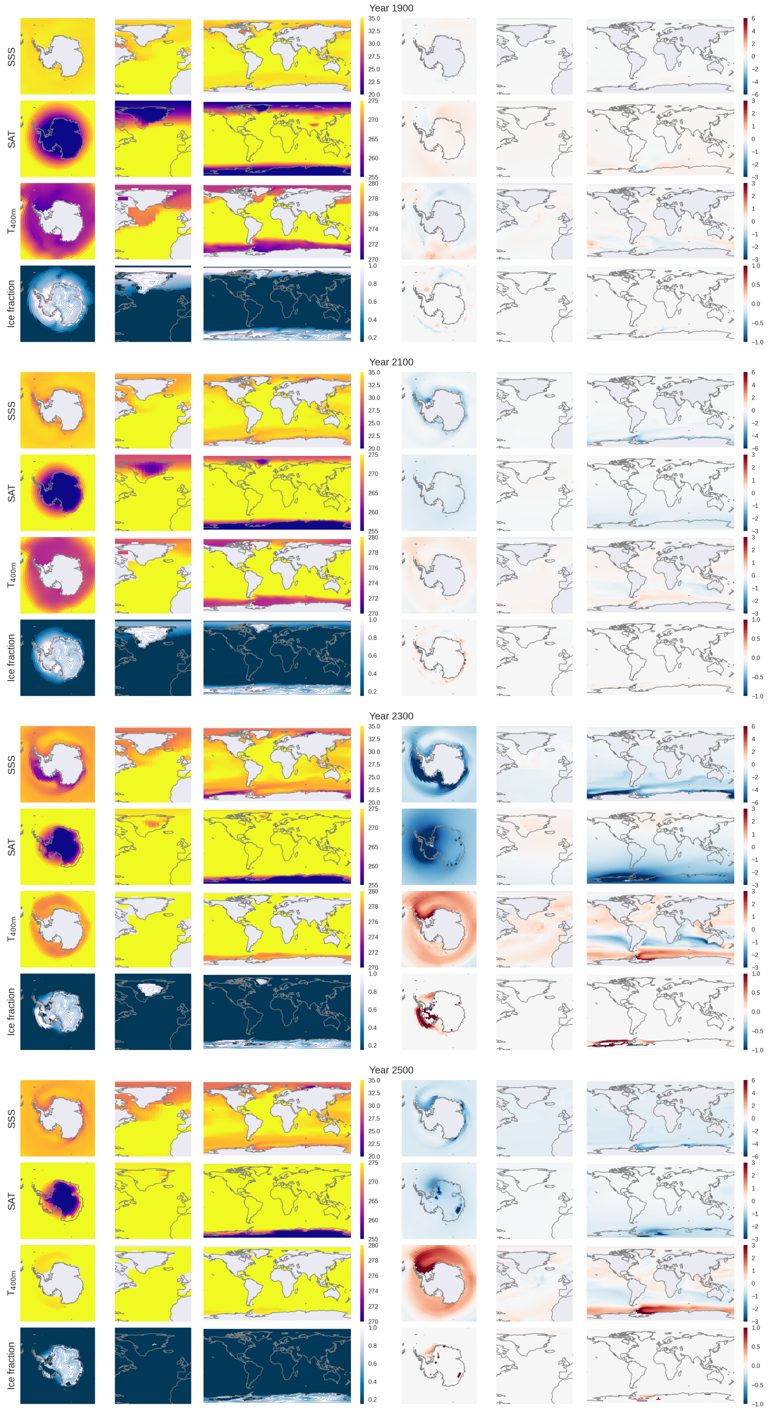

Supplementary Fig. 12: Same as Supplementary Fig. 11 but with the FWA (interactive Antarctic freshwater flux) configuration.

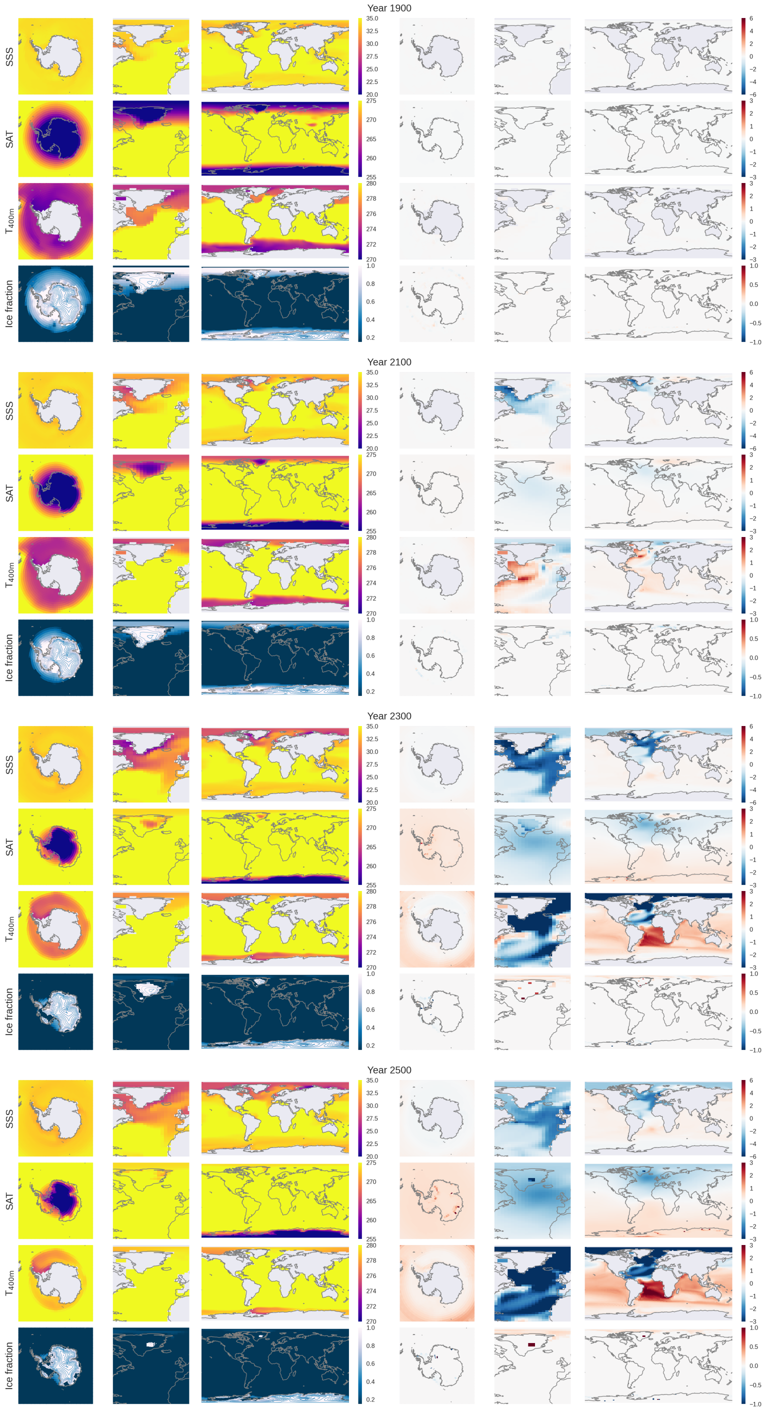

Supplementary Fig. 13: Same as Supplementary Fig. 11 but with the FWG (interactive Greenland freshwater flux) configuration.

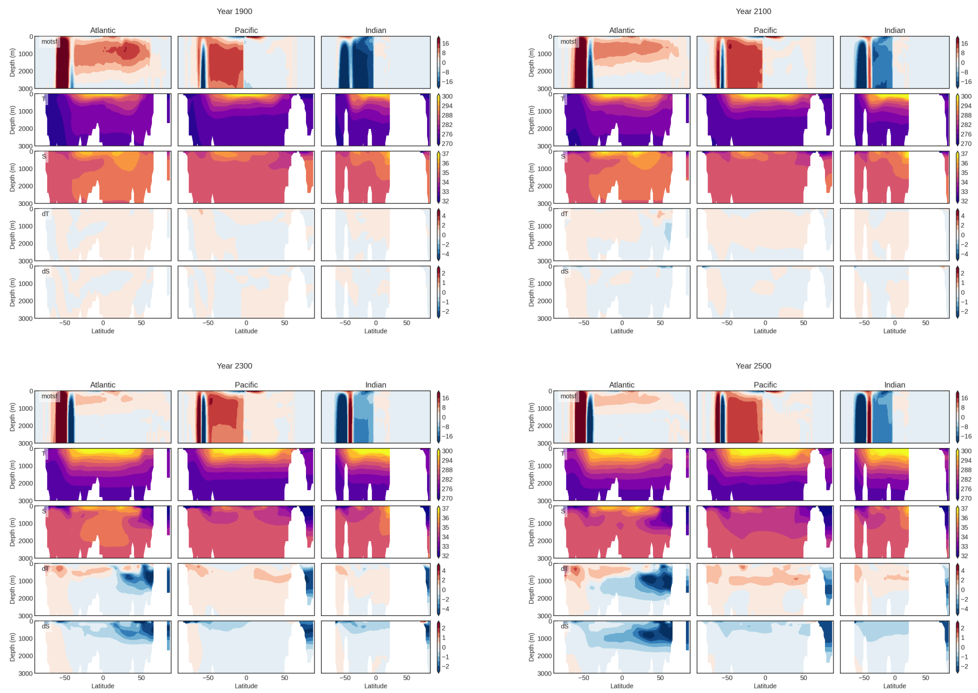

Supplementary Fig. 14: Snapshots of latitude-depth cross-sections of selected variables in year 1900, 2100, 2300, and 2500 from coupled ice sheet-climate model simulation with the FWAG (interactive freshwater flux for both ice sheets) configuration under historical-SSP5-8.5 and ECS = 5.6 °C. In each panel group, top three rows show meridional overturning stream function (motsf), ocean temperature (T), and salinity (S), and the bottom two rows show differences in ocean temperature (dT) and salinity (dS) between the simulation with interactive ice sheet freshwater flux from both ice sheets (FWAG) and that without interactive ice sheet freshwater flux. Cross-sections for temperature and salinity are taken at 34.2°W, 171°W, and 66.6°E for the Atlantic, Pacific, and Indian Ocean, respectively. Yearly evolution of these variables from 1850 to 2500 are available in Supplementary Movie 3.

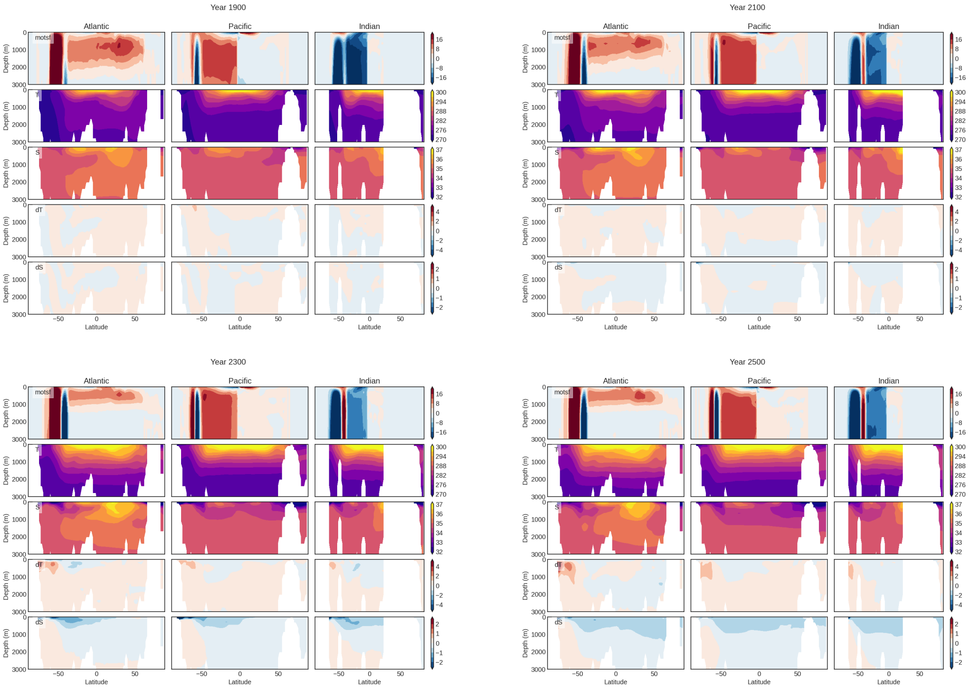

Supplementary Fig. 15: Same as Supplementary Fig. 14 but for the simulation with only interactive Antarctic freshwater flux (FWA) instead of FWAG.

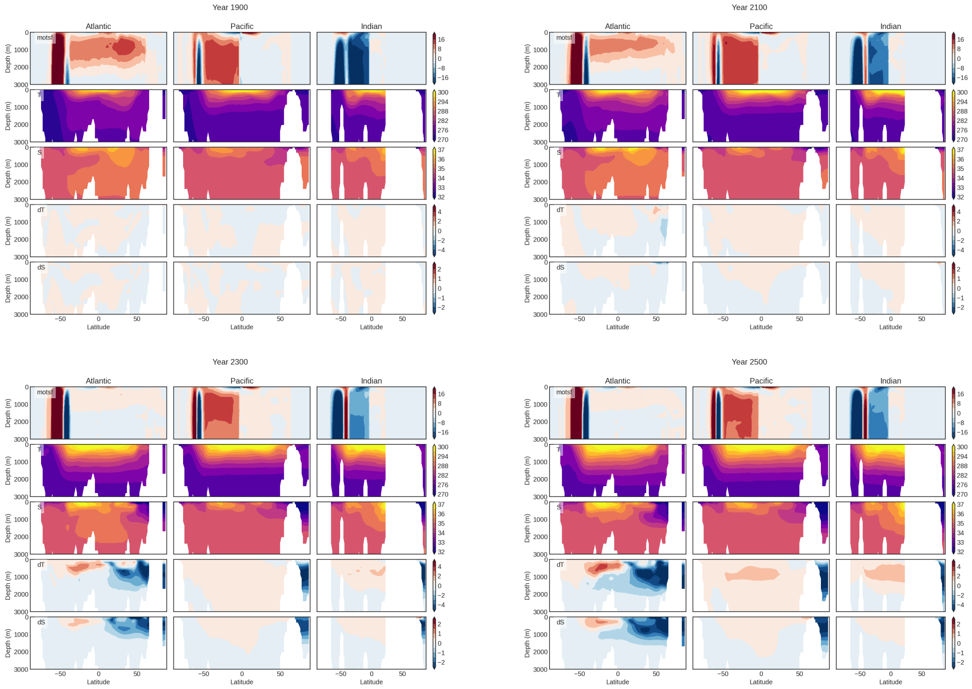

Supplementary Fig. 16: Same as Supplementary Fig. 14 but for the simulation with only interactive Greenland freshwater flux (FWG) instead of FWAG.

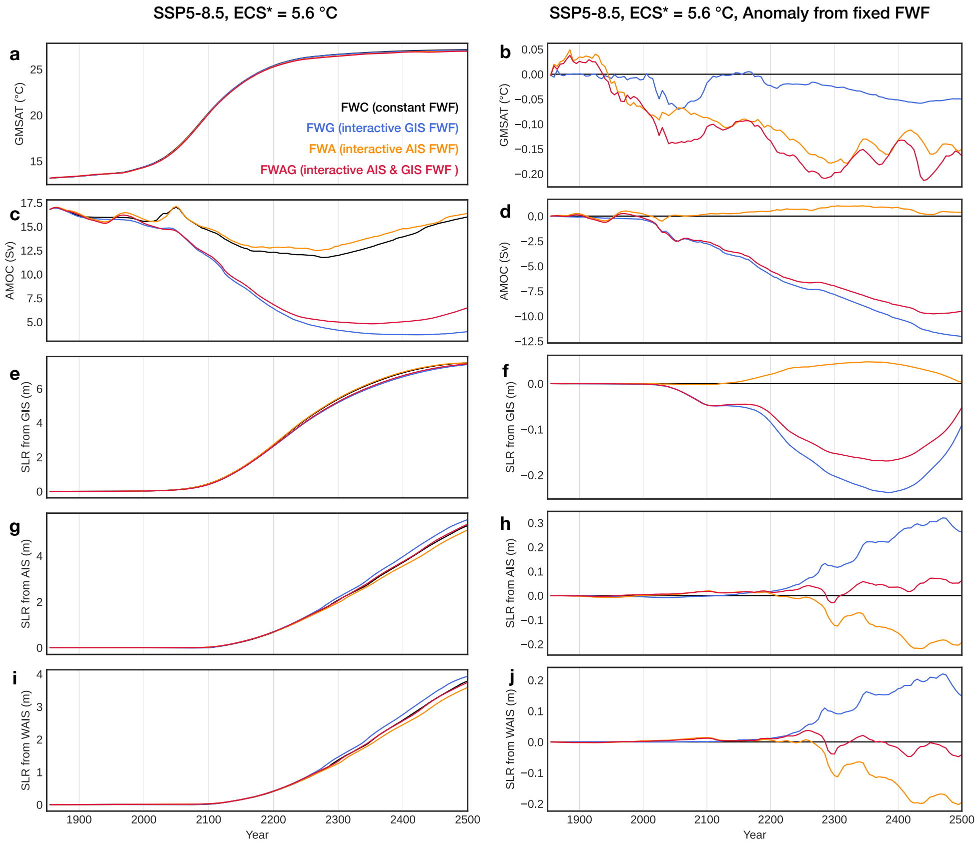

Supplementary Fig. 17: Time series of selected variables simulated by the coupled ice sheet-climate model with cliff failure turned off (Supplementary Table 1, Exp. 24). Top to bottom rows show global-mean surface air temperature (GMSAT, panels **a, b**), intensity of the Atlantic Meridional Overturning Circulation (panels **c, d**), sea level rise contributions by the Greenland Ice Sheet (panels **e, f**), by the Antarctic Ice Sheet (panels **g, h**), and by the West Antarctic Ice Sheet (panels **i, j**). The left column shows original time series and the right column anomalies relative to the the configuration without ice sheet freshwater flux (FWC).

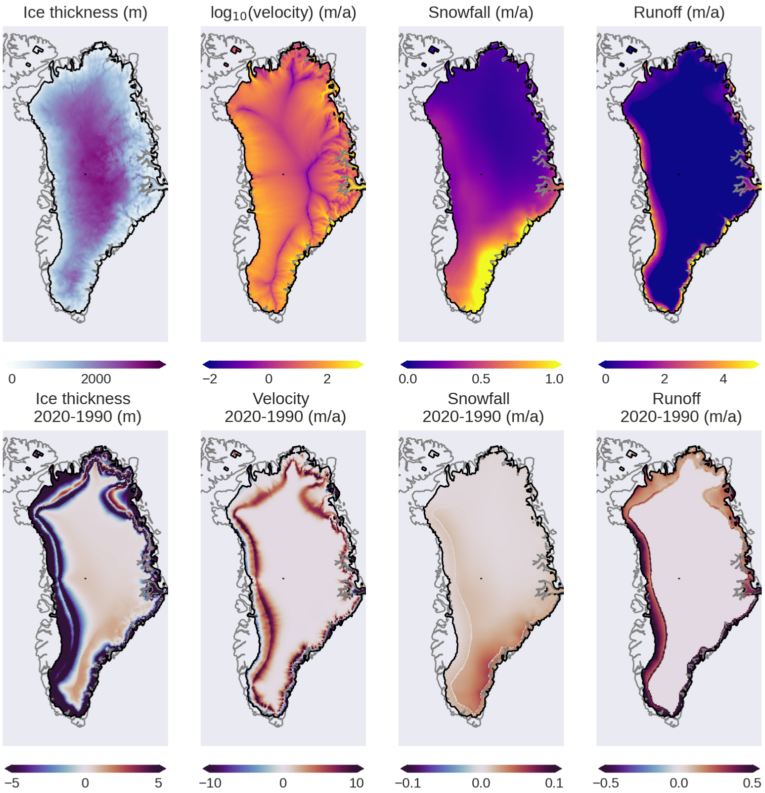

Supplementary Fig. 18: Top row: State of the Greenland Ice Sheet in year 2020 as simulated by the coupled ice sheet-climate model under historical-SSP2-4.5 scenario with an ECS of 4.0°C (10-member ensemble mean); Bottom row: Change in each variable between year 2020 and year 1990.

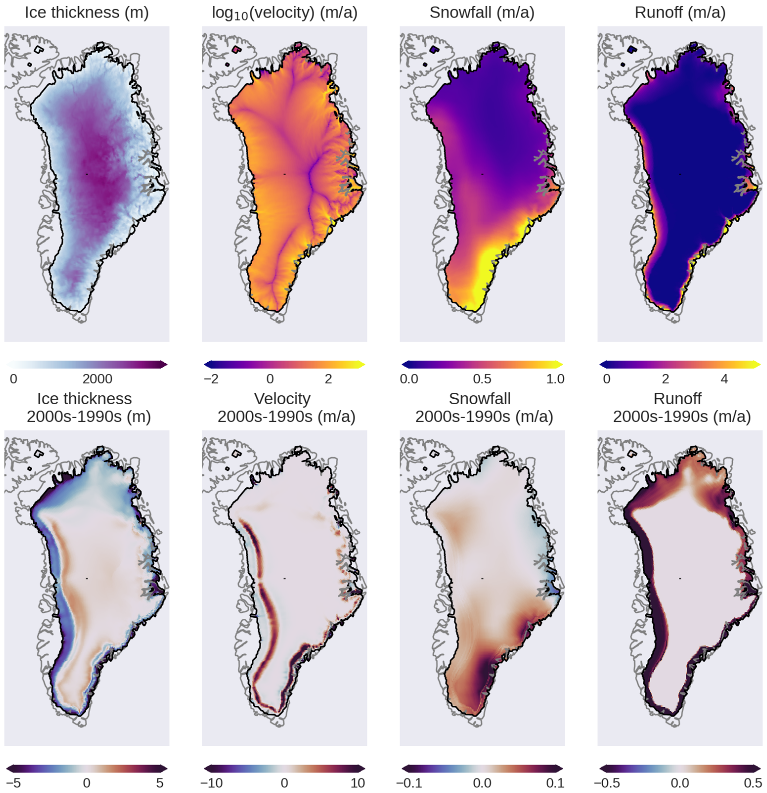

Supplementary Fig. 19: Top row: State of the Greenland Ice Sheet as simulated by the PSUICE3D ice sheet model forced by yearly climate fields from CERA20C; Bottom row: Difference in each variable between 1990s and 2000s.

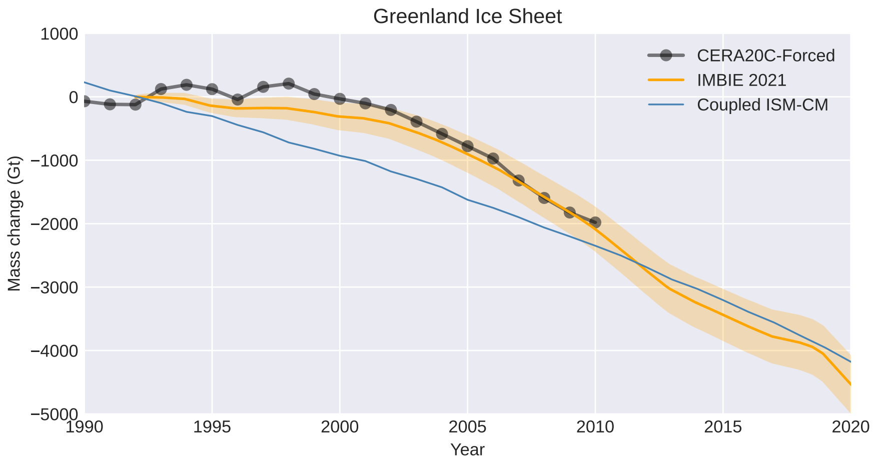

Supplementary Fig. 20: Anomaly in the Greenland Ice Sheet's mass as estimated in IMBIE 2021 and that simulated by the coupled ice sheet-climate model under historical-SSP2-4.5 scenario with an Equilibrium Climate Sensitivity (ECS) of 4.0°C.

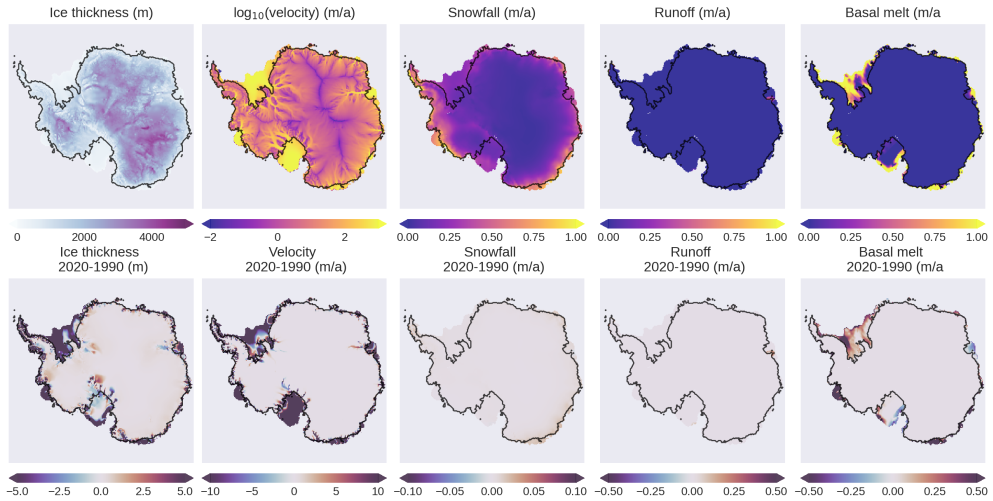

Supplementary Fig. 21: Top row: State of the Antarctic Ice Sheet in year 2020 as simulated by the coupled ice sheet-climate model under historical-SSP2-4.5 scenario with an Equilibrium Climate Sensitivity (ECS) of 4.0°C (10-member ensemble mean); Bottom row: Change in each variable between year 2020 and year 1990.

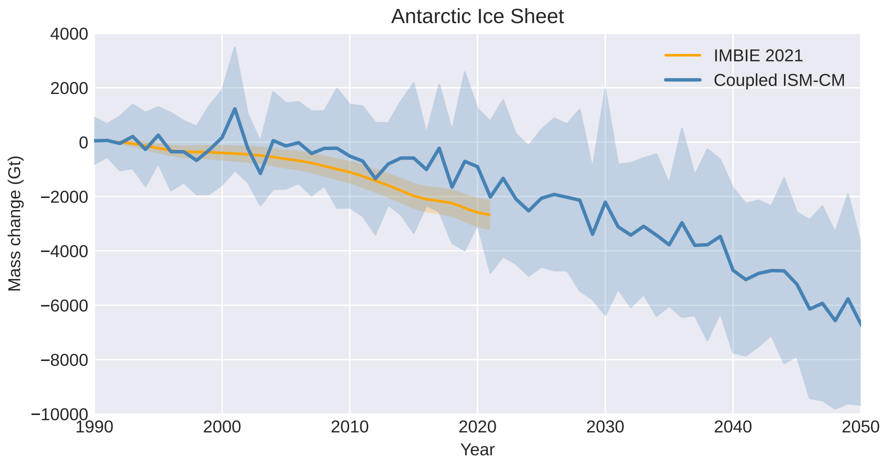

Supplementary Fig. 22: Anomaly in the Antarctic Ice Sheet's mass as estimated in IMBIE 2021 and that simulated by the coupled ice sheet-climate model under historical-SSP2-4.5 scenario with an Equilibrium Climate Sensitivity (ECS) of 4.0°C.
