# Supplementary material for: Competing climate feedbacks of ice sheet freshwater discharge in a warming world
Source: Nat Commun. 2024 Jun 18;15:5178. doi: 10.1038/s41467-024-49604-3 (PMC11189430; doi:10.1038/s41467-024-49604-3)
Supplement: Supplementary file 3 — Description of Additional Supplementary Files [file 41467_2024_49604_MOESM3_ESM.pdf]

Description of Additional Supplementary Files

Supplementary Movie 1: Animation of maps for selected variables in a moderate warming scenario (historical-SSP2-4.5 with an Equilibrium Climate Sensitivity of 4.0 °C), simulated by the coupled model with interactive ice sheet freshwater flux from both ice sheets.

Supplementary Movie 2: Animation of maps for selected variables in an intensive warming scenario (historical-SSP5-8.5 with an Equilibrium Climate Sensitivity of 5.6 °C), simulated by the coupled model with interactive ice sheet freshwater flux from both ice sheets.

Supplementary Movie 3: Animation of vertical cross-sections for selected variables in an intensive warming scenario (historical-SSP5-8.5 with an Equilibrium Climate Sensitivity of 5.6 °C), simulated by the coupled model with interactive ice sheet freshwater flux from both ice sheets.
